# Supplementary material for: A network dynamical simulation model for the study of antibiotic resistance in microbial communities
Source: Gut Microbes. 2026 Sep 19;18(1):2734703. doi: 10.1080/19490976.2026.2734703 (PMC13596919; doi:10.1080/19490976.2026.2734703)
Supplement: Supplementary file 1 [file KGMI_A_2734703_SM9053.zip › supplementary_file_1/Figures/ba_resistance_timeseries_m_5_n_100.pdf]

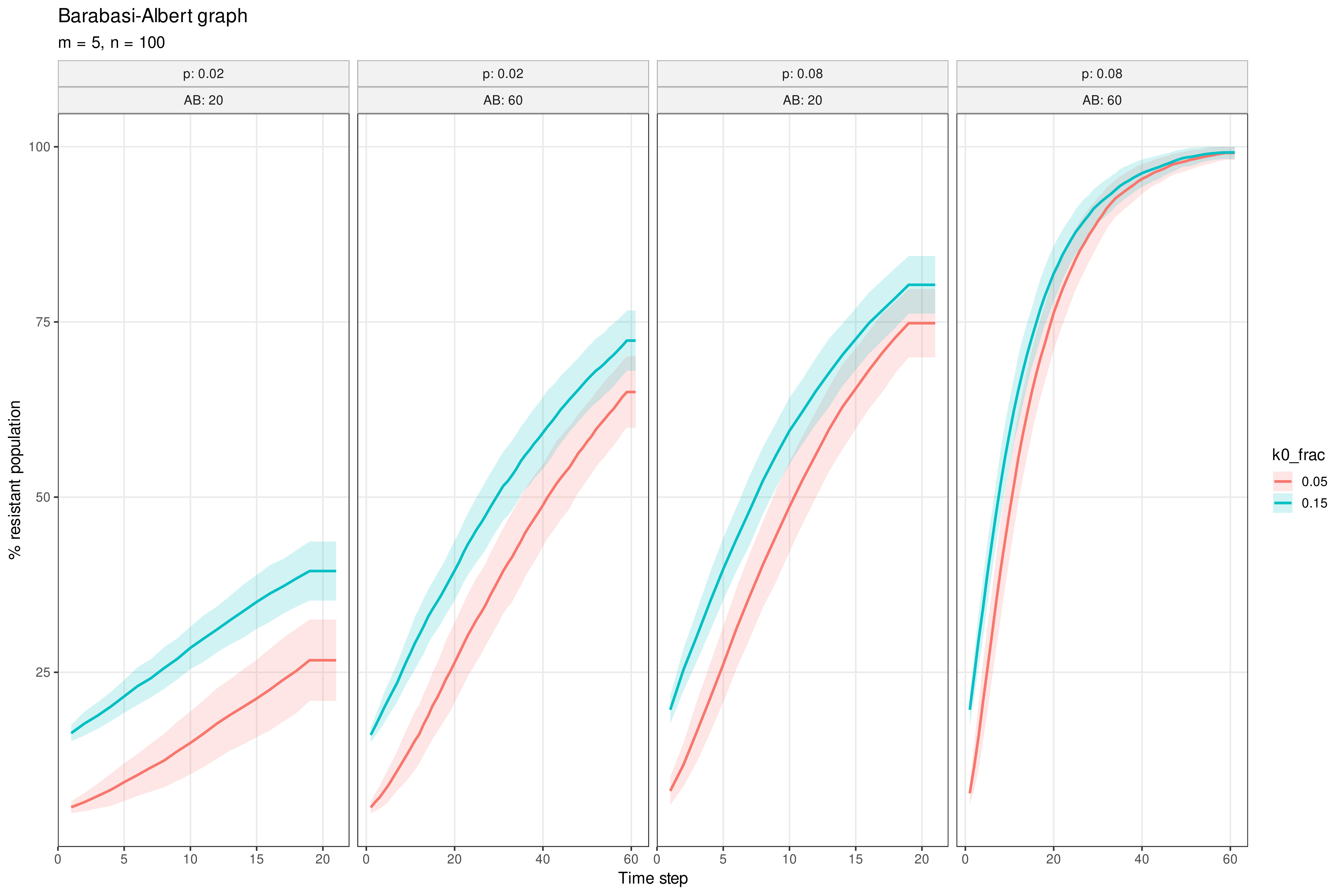
Barabasi-Albert graph
m = 5, n = 100
p: 0.02
AB: 20
p: 0.02
AB: 60
p: 0.08
AB: 20
p: 0.08
AB: 60
% resistant population
100
75
50
25
0
5
10
15
20
0
20
40
60
Time step
k0_frac
0.05
0.15
